# Nanotechnology-Based Strategies for Hair Regeneration: Mechanistic Insights and Translational Perspectives for Androgenetic Alopecia

**DOI:** 10.3390/biomedicines14030521

**Published:** 2026-02-26

**Authors:** Wenran Zhou, Rongcheng Han

**Affiliations:** 1 Beijing Hairverse Biotechnology Co., Ltd., Beijing 100192, China; wenranzhou@163.com; 2 Laboratory of Integrative Physiology, Institute of Genetics and Developmental Biology, Chinese Academy of Sciences, Beijing 100101, China

**Keywords:** androgenetic alopecia, translational nanomedicine, nanotechnogy, hair regeneration, nanomaterials, hair follicle

## Abstract

Androgenetic alopecia (AGA) is a highly prevalent and progressive disorder characterized by follicular miniaturization and dysregulation of the hair follicle microenvironment. Although minoxidil (MXD) and finasteride remain first-line therapies, their long-term efficacy is limited by poor follicular bioavailability, systemic side effects, and suboptimal patient compliance. In recent years, nanotechnology-based strategies have emerged as promising alternatives by enabling efficient follicular targeting and controlled therapeutic delivery. This review critically summarizes recent advances in nanotechnology-enabled approaches for AGA management, including nanocarrier-based formulations and nanotechnology-based microneedle systems. Beyond functioning as passive drug carriers, emerging nanoplatforms increasingly act as active modulators of the follicular niche by attenuating oxidative stress, inflammation, impaired angiogenesis, and stem cell dysfunction—key pathological drivers of AGA progression—thereby representing a conceptual shift from delivery-centered to microenvironment-remodeling strategies. To enhance translational relevance, we compare nanotechnology-based therapies with conventional treatments in terms of efficacy, safety, and clinical feasibility, and summarize representative preclinical studies, patent landscapes, and ongoing or completed clinical trials. Finally, key challenges related to safety evaluation, manufacturing reproducibility, and regulatory classification are discussed, highlighting nanotechnology as a promising framework for next-generation, mechanism-oriented AGA therapy and precision trichology.

## 1. Introduction

Hair follicles are highly dynamic and complex mini-organs embedded within the skin, characterized by a tightly orchestrated cyclical process consisting of growth (anagen), regression (catagen), and rest (telogen) phases [1,2,3,4,5]. This cyclic regeneration is governed by intricate interactions among epithelial cells, dermal papilla cells, surrounding mesenchymal tissues, immune components, and vascular networks. Precise temporal and spatial regulation of signaling pathways-including Wnt/β-catenin [6,7], Sonic hedgehog (Shh) [8,9], transforming growth factor-β (TGF-β) [10], and bone morphogenetic protein (BMP) [11] signaling-is essential for maintaining normal hair follicle homeostasis and sustaining hair shaft production [12].

Disruption of this finely tuned hair cycle leads to a spectrum of alopecia disorders, among which AGA represents the most prevalent and clinically significant form [13,14,15,16]. AGA is a chronic, progressive condition characterized by the gradual miniaturization of hair follicles, shortening of the anagen phase, and prolongation of telogen, ultimately resulting in thinner, shorter, and less pigmented hairs [17,18,19,20,21,22]. Epidemiological studies indicate that AGA affects more than 50% of men and a substantial proportion of women over the course of their lifetime, with incidence increasing with age and genetic predisposition [23,24,25]. Beyond its physical manifestations, AGA imposes considerable psychosocial burdens, including diminished self-esteem, increased anxiety, and reduced quality of life, underscoring the need for effective and well-tolerated therapeutic interventions.

Currently, conventional pharmacological management of AGA predominantly relies on topical MXD and oral finasteride, both of which have received regulatory approval and are widely used in clinical practice [26,27,28,29]. MXD is thought to promote hair growth primarily through vasodilation, potassium channel activation, and indirect stimulation of dermal papilla cell function, whereas finasteride exerts its effects by inhibiting type II 5α-reductase, thereby reducing dihydrotestosterone (DHT) levels in the scalp [30,31,32]. Despite their established efficacy, clinical outcomes remain highly variable, and long-term treatment is often required to maintain therapeutic benefits [33,34].

Importantly, these conventional therapies suffer from several intrinsic limitations, including insufficient follicular bioavailability, rapid clearance from the scalp, and poor patient adherence due to frequent application or systemic exposure [35]. Topically applied drugs face formidable barriers such as the stratum corneum and uneven follicular penetration, while systemic administration of antiandrogens may lead to undesirable side effects that limit widespread acceptance [36]. Consequently, there exists a substantial unmet clinical need for innovative therapeutic strategies capable of achieving efficient, localized follicular targeting, sustained drug retention, and minimal off-target effects. Addressing these challenges has catalyzed growing interest in advanced drug delivery platforms—particularly nanotechnology-based approaches—as next-generation solutions for AGA treatment.

Nanotechnology offers unprecedented opportunities to overcome the formidable anatomical and physiological barriers presented by the skin and hair follicle, which have long limited the efficacy of conventional topical and systemic therapies [37,38,39,40,41]. The stratum corneum, complex extracellular matrix, and dynamic immune surveillance collectively restrict drug penetration and retention, resulting in suboptimal exposure of therapeutics at critical follicular compartments [42]. By contrast, nanoscale delivery systems can be rationally engineered with tunable size, surface charge, and chemical functionality to enhance penetration, prolong residence time, and achieve controlled release within the pilosebaceous unit [43,44,45,46].

Notably, the unique architecture of the hair follicle provides a privileged and biologically relevant gateway for nanomaterials. The follicular infundibulum and sebaceous duct function as a natural reservoir capable of selectively accumulating nanoparticles, thereby enabling localized drug storage and sustained delivery directly to the vicinity of dermal papilla cells and hair follicle stem cell niches [47]. This follicular targeting effect allows nanotechnology-based systems to bypass the stratum corneum barrier while minimizing systemic exposure, a critical advantage for long-term management of AGA [48,49,50,51,52]. Furthermore, the physicochemical properties of nanomaterials can be tailored to exploit follicle-specific features such as sebum affinity, cyclic hair movement, and size-dependent penetration, collectively enhancing therapeutic precision.

Beyond serving as passive carriers, emerging nanotechnology-enabled platforms increasingly function as active modulators of the follicular microenvironment [53,54,55,56]. Advanced nanomaterials have been designed to regulate oxidative stress, promote angiogenesis, modulate inflammatory responses, and activate hair follicle stem cells, thereby addressing multiple pathogenic drivers of AGA simultaneously [56,57,58,59,60]. Such multifunctional capabilities distinguish nanotechnology-based approaches from traditional formulations and align with the growing recognition that effective hair regeneration requires coordinated modulation of both cellular and microenvironmental cues.

Herein, we present current knowledge and emerging trends in nanotechnology-based strategies for hair regeneration, with a particular emphasis on AGA. We critically examine nanocarrier design principles, follicular targeting mechanisms, and nanotechnology-based therapeutic modalities, while highlighting mechanistic insights and translational challenges. By integrating recent experimental advances with clinical perspectives, this review aims to provide a comprehensive framework for the rational development of next-generation nanotechnology-enabled therapies for AGA. Unlike previous reviews that primarily emphasize the delivery of FDA-approved drugs, this article focuses on the rational design of multifunctional nanomaterials (e.g., nanozymes) that actively modulate the oxidative and inflammatory niches within the hair follicle.

## 2. Pathophysiology of AGA

**Figure 1:**
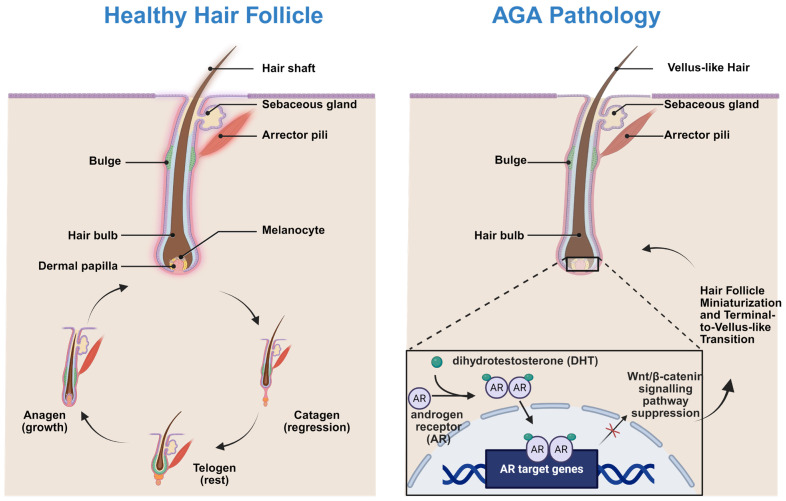
Schematic overview of hair follicle anatomy, cycling dynamics, and key pathological alterations associated with AGA. Created in Biorender. W. Zhou. (2026) https://app.biorender.com/illustrations/69636b281654f4e8f8518cc9 (accessed on 13 January 2026).

The pathogenesis of AGA is multifactorial (Figure 1), involving genetic susceptibility, androgen signaling, chronic inflammation, oxidative stress, and microvascular impairment [61,62,63,64]. Central to AGA progression is DHT, which binds androgen receptors in dermal papilla cells and induces transcriptional programs that suppress Wnt/β-catenin signaling and anagen maintenance [65].

Beyond androgen signaling, increasing evidence highlights the contribution of perifollicular inflammation, elevated reactive oxygen species (ROS), and impaired angiogenesis to follicular miniaturization [66,67,68]. Oxidative stress accelerates cellular senescence within dermal papilla cells and disrupts hair follicle stem cell niches. These insights underscore the necessity for multifunctional therapeutic approaches capable of simultaneously modulating multiple pathological pathways [69,70].

## 3. Nanocarrier-Based Drug Delivery Systems for AGA

**Figure 2:**
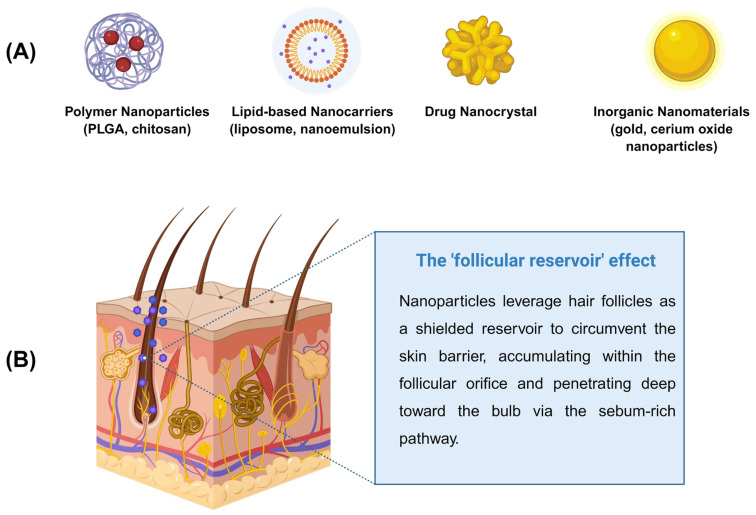
Classification of nanocarrier-based delivery systems for AGA (**A**) and their follicular targeting and penetration mechanisms (**B**). Created in Biorender. W. Zhou. (2026) https://app.biorender.com/illustrations/69635c141654f4e8f8418550 (accessed on 13 January 2026).

**Figure 3:**
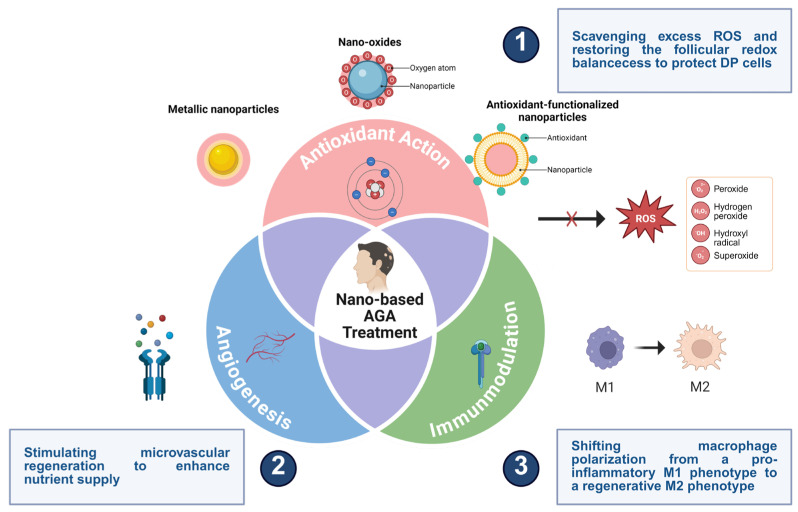
Nanotechnology-enabled multimodal remodeling of the follicular microenvironment through regulation of oxidative stress, angiogenesis, and perifollicular inflammation. Created in Biorender. W. Zhou. (2026) https://app.biorender.com/illustrations/696383e2cf65f44b8a3ca108 (accessed on 14 January 2026).

Nanocarrier systems have been extensively explored to enhance the follicular delivery and therapeutic efficacy of AGA drugs [71,72]. Polymeric nanoparticles, lipid-based carriers, nanocrystals, and inorganic nanomaterials represent the major classes investigated to date (Figure 2, Table 1 and Figure 3) [73,74,75,76].

Polymeric nanoparticles fabricated from PLGA, chitosan, or hyaluronic acid (HA) improve drug stability and enable sustained release within hair follicles [77]. Lipid nanoparticles and nanoemulsions enhance skin penetration and follicular retention through sebum affinity [78]. Drug nanocrystals maximize loading capacity and dissolution rates, allowing dose reduction and improved patient compliance [79,80]. Collectively, these nanocarriers significantly outperform conventional formulations in follicular targeting efficiency [78,81,82,83,84,85].

**Table 1:** Representative nanocarrier systems for AGA treatment.

Nanocarrier Class	Example Materials	Therapeutic Mechanism/Drug Delivered	Size (nm)	Zeta Potential (mV)	Application	Key Advantages for AGA	References
Polymeric Nanoparticles	Methylcellulose	MXD	90–300	NA	In vivo AGA-induced C57BL/6 mouse model	Enhance drug aggregation and expressions of hair-growth factors in hair bulbs No skin stimulation	[86]
	Chitosan	MXD	235.5 ± 99.9	+38.6 ± 6.0	In vitro porcine ears skin permeation test	Sustained drug release Increased drug permeation into hair follicles	[82]
	PLGA	finasteride	316.5 ± 14.4	NA	In vitro polydimethylsiloxane membrane permeation test	Encapsulation efficiency 79.49% ± 0.47%	[87]
	HA-PLGA	MXD	243 ± 44.5	NA	In vitro rat skin permeation test	Higher skin permeability Uptake by hair follicle dermal papillary cells	[80]
	Poly-ε-caprolactone	Latanoprost	97.8 ± 1.2	−30.1 ± 1.8	In vitro porcine ears skin permeation test	Stable storage for 90 days Improved drug accumulation into hair follicles	[88]
	Methyl-β-cyclodextrin 10% Polyvinyl Pyrrolidone K30	Rosuvastatin	218	NA	In vivo hair loss Albino rat model	Sustained drug release Activation of epithelial stem cells of hair follicle	[89]
	Poly-(q-caprolactone)-block-poly(ethyleneglycol)	MXD	40–130	NA	In vivo skin retention test	Penetrated mainly via hair follicles routes	[90]
	Dipalmotyl (DPPC)-PLGA	Quercetin	339 ± 1.6	−32.6 ± 0.51	In vivo alopecia-induced rat models	Entrapment efficiency 78% ± 5.5% Uptake by hair follicles Inhibit hair follicle cells apoptosis in vivo	[91]
	Ethyl cellulose	α-Mangostin	436.0 ± 11.5	NA	Therapeutic effect study in 10 acne patients	Sustained release in human synthetic sebum Excellent hair follicle entrapment	[92]
	PEG5K-b-oligo (DTO-SA)-b-PEG5K	Adapalene	64.7–81.6	NA	In vitro human cadaver and porcine ear skin permeation test	Increased drug accumulation in hair follicles	[93]
	Poly(amidoamine)	Adapalene	256 ± 12	19.0 ± 3.1 (0.05%) 27.6 ± 2.3 (0.07%)	In vitro abdominal porcine skin permeation test	Increased drug accumulation in hair follicles and skin.	[94]
	Eudragit^®^ L100	Dexamethasone	303.1 ± 5.5	NA	In vitro porcine skin permeation test	pH-sensitive Significant transfollicular penetration	[95]
	poly- ε-caprolactone	Adapalene	107.5 ± 8.19	−13.1	In vitro full-thickness human skin permeation and distribution test	Preferential targeting to PSU	[96]
	Chitosan HA	Clindamycin	362 ± 19 417 ± 9	27.7 ± 0.9 −30.2 ± 2.7	In vitro skin penetration test using intact skin porcine, skin with the PSU artificially blocked, and sebaceous skin	Enhanced targeted delivery to pilosebaceous structures	[97]
	*Delonix* polymer	Isotretinoin	230 ± 10	−67 ± 3	In vitro pig ear skin permeation test	Significant follicular targeting Function as follicular drug reservoir	[98]
	Polylactic acid (PLA)	Cyclosporin A	152.2 ± 5	−16 ± 0.2	In vitro porcine skin permeation test	Increased skin permeation/hair follicles accumulation	[99]
	Poly-(ɛ-caprolactone)-lipid	Dutasteride	199.0 ± 0.5	− 13.6 ± 0.6	In vitro porcine’s ear skin permeation test	Fivefold increase in hair follicles targeting	[100]
	D-α-tocopheryl polyethylene glycol succinate diblock copolymer	Adapalene	4–12	NA	In vitro full-thickness porcine and human skin permeation test	Preferential accumulation in the follicular orifice	[101]
	Pluronic^®^ F127	Benzoyl peroxide	24.8–25.9	−2 to −13	In vitro porcine skin permeation test	Drug deposition in the follicular pathway	[102]
	Clove oil Kolliphor^®^ P188	MXD	10	NA	In vitro follicular drug penetration test	Controlled drug release Twenty-sixfold drug penetrated into hair follicles	[103]
	Eucalyptol Oleic Acid	MXD	29.6 ± 3.1 19.5 ± 1.3 8.0 ± 0.5 12.4 ± 0.1	NA	In vitro full-thickness excised human skin permeation test	Promoted drug retention in deeper skin layers Greater hair follicle penetration	[104]
	Soya lecithin Polyethylene glycol 600	FIN	195.2 ± 9.43	−7.61 ± 1.35	In vivo AGA-induced Swiss albino mouse model	Increased hair diameter and length Restored the follicle station Be safe and stable for more than 90 days	[105]
	Poly (ethylene oxide)-block-poly(ε-caprolactone) Lecithin	Luteolin	290	NA	In vivo alopecia-induced C57BL/6 mouse model	Stability for long-term storage Hair growth-promotion activity	[106]
	Medium chain oil Span 80	Cedrol	14.26 ± 0.16	NA	In vivo alopecia-induced C57BL/6 mouse model	Improved drug solubility Increased growth rate of hair follicles	[107]
Lipid-based Carriers	Stearic acid Oleic acid	MXD	281.4 ± 7.4	−32.9 ± 1.23	In vitro rat skin permeation test	Drug entrapment efficiency 92.48% ± 0.31% Promoted hair follicles retention	[108]
	Phospholipid Cholesterol	MXD, Tretinoin	149.33 ± 1.4	7.74 ± 0.22	In vitro rat skin permeation test	Promoted hair layers retention	[109]
	Squalene Precirol^®^ Anti-platelet-derived growth factor	MXD	236.0 ± 3.3 194.5 ± 4.7	−43.8 ± 0.9 −45.5 ± 0.6	In vivo skin permeation test	Ameliorated follicular uptake Promoted proliferation of dermal papilla cells Up regulation of hair regeneration related factor	[110]
	Squalene	Diphencyprone	236.3 ± 3.2	−52.8 ± 4.7	In vivo nude mouse dorsal skin permeation test	Improved drug targeting to follicles	[111]
	Olive oil Transcutol^®^ Tween 80	Spironolactone	215.6 ± 20.4	−18.7 ± 0.92	In vitro skin permeation test	Entrapment efficiency 87.36% ± 3.34% Deliver the NLCs within the follicles	[112]
	Stearic acid	Dutasteride	187.6 ± 7.0	−18 ± 0.9 (uncoated) 25.8 ± 1.1 (coated)	In vitro porcine skin permeation test	Entrapment efficiency 97.8% ± 0.68% Promoted penetration in the hair follicular region	[113]
	Lauric acid Chitosan	Dutasteride	184.2 ± 2.9	−18 ± 2.3 (uncoated) 24.8 ± 2.1 (coated)	In vitro porcine skin permeation test	Physically stable for 180 days Enhanced cell proliferation of human dermal papilla cells	[114]
	Stearic acid Cholesterol Triolein	Cyproterone acetate	300	−35 ± 0.5	In vivo hamsters skin permeation test	Enhanced accumulation in hair follicles Increased drug accumulation in dermis and epidermis	[115]
	Palmitostearate Evening primrose Olive Soybean Bitter almond	Melatonin	683 ± 27.08 307 ± 18.31 307 ± 3.68 303 ± 16.24	−17. 2 ± 0.53 −15.1 ± 0.22 −6.6 ± 0.14 −14.6 ± 0.78	Therapeutic effect study in 40 male AGA patients	Increased hair density and thickness	[116]
	Precirol^®^ Oleic acid	Arginine	87.34	−24.6	In vivo hamsters skin permeation test	Increased accumulation in the hair follicles Accelerate new hair follicle growth	[117]
	Buriti oil Ceramides	17-α-estradiol	96 ± 15	−17 ± 6	In vivo human skin permeation test	Encapsulation efficiency 99.6% ± 0.3% Physical stability for 42 d Accumulation in the hair follicle	[118]
	Glyceryl distearate Glyceryl monostearate Tween 80 or Span 65	Adapalene	300.3 ± 1.45	−21.3 ± 0.07	Clinical study in 15 acne vulgaris patients	Sustained drug release Improvement in pilosebaceous follicles	[119]
	Stearic acid Oeic acid	Clindamycin phosphate	400 ± 14	−48.9 ± 0.7	In vivo skin permeation on porcine skin	Increased accumulation into hair follicles openings	[120]
	Precirol ATO-5^®^ Span 80	Flutamide	192 ± 13	NA	In vivo skin permeation and hair growth test	Good stability for two months Higher accumulation in the hair follicles	[121]
Transferosome	FIN Finasteride	Phospholipon 90 G Span 65	299.6 ± 45.6 171.0 ± 5.6 197.4 ± 29.1	NA	In vivo rat skin permeation test	Enhanced drug permeation in skin layer	[122]
	MXD Caffeine	Polysorbate 20 Polysorbate 80	NA	NA	In vivo AGA-induced rat model	Enhanced hair length	[123]
Ethosome	Cryptotanshinone	Soybean phosphatidycholine Ethanol	69.1 ± 1.9	NA	In vivo anti-acne effect in rabbit model	Increased anti-acne effect	[124]
Liquid crystal nanocarrier	MXD	Monoglycerides Phospholipids Poloxamer 407	82 ± 1	−57 ± 3	In vivo hair regrowth efficacy test on rats	Selective delivery to pilosebaceous follicle	[125]
Nanozyme-integrated dissolving microneedles (Ce-MNs)	Core: Ceria nanozymes (CeNZs) modified with DSPE-mPEG_2000_ Needle matrix: Hyaluronic acid (HA, Mw < 10 kDa) Backing: Polyvinylpyrrolidone (PVP-K90)	Dual-mode regulation of perifollicular microenvironment ROS scavenging (CAT- and SOD-mimic activities, HORAC) Angiogenesis promotion (mechanical stimulation-induced VEGF upregulation) Delivered: CeNZs (~8.65 μg Ce per patch)	Hydrophobic CeNZs: ~3 nm (TEM) PEGylated CeNZs: ~10 nm (hydrodynamic diameter)	NA	In vivo AGA-induced C57BL/6 mouse model	Superior treatment efficiency: Faster onset of telogen-to-anagen transition vs. MXD with lower administration frequency (5 applications vs. daily topical) Effective transdermal delivery: Bypasses stratum corneum to deliver CeNZs to 200–300 μm depth (hair follicle residence) High safety profile: Biocompatible with no significant/irreversible skin damage; epidermal thickening is reversible by day 28 Comparable efficacy: Achieves similar hair diameter, density, and coverage as MXD with intact hair scales	[126]
Finasteride–peptide nanocomplexes	Peptide with hydrophobic blocks (PepWL, PepW4) coassembled with Finasteride	Finasteride: 5α-reductase inhibitor, reduces DHT. CPPecp peptide: Skin/cell-penetrating and anti-inflammatory. Synergy: Promotes dermal papilla cell viability and hair regeneration.	NC-WL: 57.7 ± 7.0 nm NC-W4: 133.5 ± 17.3 nm	NA	In vivo C57BL/6 mouse model	Carrier-free: Peptide is both delivery vehicle and therapeutic. Synergistic: Enhances efficacy with anti-inflammatory action. Low systemic risk: Topical; finasteride dose ~1/40 of oral standard. Effective: Hair growth comparable to 5% minoxidil; accelerates catagen-to-anagen transition.	[127]
Hyaluronic acid liposome (HL) composite	Soybean phosphatidylcholine (Lecithin) Cho-PEI/NONOate (NO donor) MXD (Mi) Hyaluronic acid (HA)	Vasodilation: NO→cGMP pathway induces capillary dilation, accelerating blood flow to enhance Mi penetration Prolonged retention: Liposome structure extends Mi residence time in skin Anti-inflammation: Downregulates IL-6 and TGF-β1 in follicles Angiogenesis: Upregulates VEGF expression Stem cell activation: Upregulates β-catenin, MMP3, Ki67, and PCNA to induce follicle regeneration	Hydrated: ~350–520 nm (<500 nm) Dry (TEM): ~200 nm	HL@Mi: −12 mV HL@Mi/NONOate: −24 mV	In vivo AGA-induced C57BL/6 mouse model	Synergistic multimodal therapy: Combines gas molecule (NO) with drug (Mi) for enhanced efficacy Enhanced penetration: NO-induced vasodilation significantly improves transdermal Mi delivery compared to conventional tinctures Prolonged action: Extended drug retention in skin improves bioavailability Microenvironment regulation: Simultaneously addresses vascular insufficiency, inflammation, and stem cell activation Superior biocompatibility: Avoids skin irritation (dryness, peeling, crystallization) caused by ethanol/propylene glycol in commercial MXD formulations Comparable efficacy: Achieves hair regrowth comparable to MXD with reduced side effects and inflammation	[128]

## 4. Nanotechnology-Based Microneedle Systems for Transdermal Follicular Delivery

**Figure 4:**
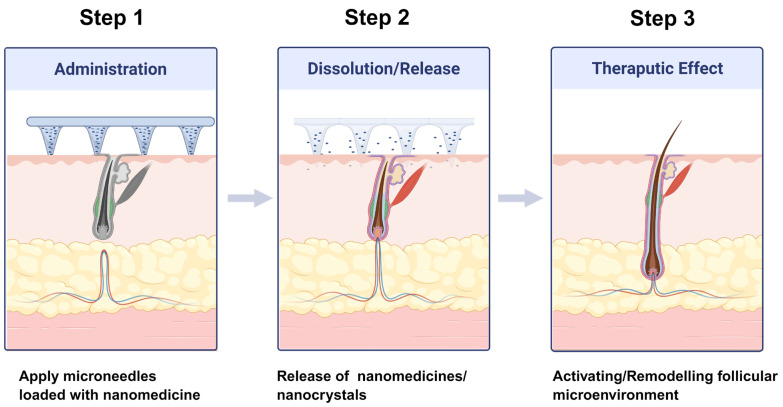
Design strategies, structural configurations, and working principles of nanotechnology-based microneedle systems for transdermal follicular delivery. Created in Biorender. W. Zhou. (2026) https://app.biorender.com/illustrations/6963a335e9af035c7186d171 (accessed on 14 January 2026).

Microneedle (MN) technology provides a minimally invasive approach to bypass the stratum corneum and directly access follicular and dermal compartments (Figure 4) [76,129,130,131,132]. The integration of nanotechnology into MN systems has further expanded their functional capabilities.

Dissolving MNs loaded with drug nanocrystals or nanoparticles enable precise dosing and sustained release. Advanced designs incorporating nanozymes or growth factor-loaded nanoparticles actively regulate oxidative stress, angiogenesis, and inflammation [133,134,135]. Such multifunctional MN systems have demonstrated superior hair regrowth efficacy in preclinical AGA models compared with conventional topical therapies. For instance, Zhang et al. reported a machine learning-guided identification of a highly efficient MnPS_3_-based SOD mimic and its microneedle patch for the treatment of AGA, which alleviates oxidative stress in hair follicles and promotes superior hair regeneration compared with MXD at a reduced application frequency [133]. More recently, Xing et al. developed a near-infrared light-triggered nitric oxide (NO)-releasing HA hydrogel (Gel@L-Arg) that enables on-demand NO generation to promote angiogenesis, repair dermal papilla cells, regulate inflammation and androgens, and effectively treat AGA [135].

Recent preclinical investigations have significantly advanced the design of nano-enabled MN systems for AGA, transitioning from simple physical conduits to ‘smart’ responsive platforms. For instance, dissolvable HA microneedles have been successfully integrated with lipid-based nanocarriers to encapsulate both MXD and finasteride [136,137]. These systems demonstrate a synergistic effect, where the MNs bypass the stratum corneum and the nanocarriers ensure sustained, deep-follicle drug release, resulting in a faster telogen-to-anagen transition in murine models.

Beyond drug delivery, a novel trend involves the use of bioactive nanostructures within MN arrays. Bimetallic nanozymes (e.g., Ni-Cu) delivered via MNs have shown remarkable efficacy in scavenging ROS and remodeling the oxidative niche, thereby protecting dermal papilla cells from senescence [60]. Furthermore, exosome-integrated hydrogel microneedles represent a cutting-edge cell-free therapy; these systems allow for the localized, spatiotemporal release of growth factors and miRNAs, promoting robust angiogenesis around the hair follicle [138,139,140]. More recently, stimuli-responsive MNs, such as pH-sensitive polymeric nanoparticles [141,142] or light-triggered gold nanostructures [143,144], have been developed to achieve ‘on-demand’ therapeutic release [145,146], offering a highly precise approach to managing the fluctuating inflammatory states of the AGA scalp.

## 5. Nanotechnology-Based Remodeling of the Hair Follicle Microenvironment

Hair regeneration is critically dependent on the follicular microenvironment, including redox balance, vascular support, immune status, as well as stem cell activity [147,148,149,150]. Beyond acting as passive carriers, some nanomaterials also possess the capability to actively remodel the local microenvironment [60].

Previous studies have demonstrated that antioxidant nanomaterials, such as polydopamine nanoparticles and ceria nanozymes, effectively scavenge excess ROS and restore redox homeostasis [56]. Pro-angiogenic nanocarriers delivering VEGF or exhibiting intrinsic angiogenic activity enhance perifollicular blood supply. Additionally, nanomaterials modulating macrophage polarization and inflammatory signaling contribute to a regenerative follicular niche conducive to sustained hair growth. Yang et al. developed PDA@QLipo, a quercetin-encapsulated nanosystem designed to promote hair regeneration. This platform functions by remodeling the perifollicular microenvironment and effectively mitigating localized oxidative stress. PDA@QLipo exhibits dual functions of ROS scavenging and angiogenesis promotion. In vivo, roller-microneedle-assisted delivery effectively rejuvenated the compromised perifollicular niche, enhancing cell proliferation, accelerating follicle renewal, and restoring hair growth. Notably, PDA@QLipo achieved a higher hair regeneration coverage (92.5%) than MXD (87.8%) with reduced dosing frequency, highlighting its potential for clinical AGA therapy [56]. More recently, a dissolvable microneedle system co-loaded with nickel–copper nanozymes demonstrating remarkable SOD-like and CAT-like activities and MXD synergistically remodels the hair follicle microenvironment via ROS scavenging and mechanostimulation-enhanced angiogenesis, achieving superior hair regeneration and vascularization compared with MXD alone [60]. In AGA mouse models, this system enhanced hair regeneration coverage to 93.7% (vs 85.1% for MXD alone), increased Ki67+ cell proliferation by 1.9-fold, and significantly thickened regenerated hair diameter. Additionally, this system reduced ROS levels by 2.3-fold and increased CD31+ vascular density by 40%, markedly improving the microenvironment.

To elucidate how nanotechnology actively remodels the hair follicle niche, it is essential to distinguish direct material–cell interactions from conventional drug-mediated effects. Metallic nanozymes, such as Ni-Cu bimetallic nanoparticles, exhibit intrinsic SOD and CAT mimetic activity. Upon internalization by dermal papilla cells, they efficiently scavenge ROS, suppressing p38 MAPK-mediated overexpression of DKK-1, stabilizing β-catenin, and promoting transcription of hair-growth genes like AXIN2 and LEF1. Unlike MXD, which indirectly stimulates hair growth via vasodilation, these nanotechnology-based interventions directly modulate DPC signaling, maintaining the anagen state through catalytic bioactivity. This paradigm shift highlights a transition from passive drug carriers to active, signaling-modulatory bionanomaterials for AGA therapy.

Beyond manual screening of catalytic materials, the integration of artificial intelligence (AI) and machine learning has emerged as a transformative approach to predict the enzyme-mimetic activities of complex nanozymes. By optimizing atomic configurations and surface strain via AI-driven high-throughput screening, researchers can now design next-generation nanozymes with multi-enzyme activities (e.g., mimicking SOD, CAT, and POD simultaneously) to precisely counteract the multifaceted oxidative damage in the AGA follicular niche.

## 6. Safety, Toxicity, and Regulatory Considerations

The clinical translation of nanotechnology-based AGA therapies requires rigorous evaluation of safety, toxicity, and regulatory compliance [151,152]. Critical factors include nanoparticle size, surface chemistry, biodegradability, and cumulative scalp exposure [153]. While short-term biocompatibility is generally favorable, the long-term safety of repeated or chronic exposure—particularly regarding nanoparticle accumulation, immunogenicity, and off-target interactions—remains incompletely understood.

Although the follicular route enables targeted delivery, it may also facilitate systemic translocation, raising specific concerns for inorganic nanomaterials. Metal-based nanozymes, such as cerium oxide (CeO_2_) or gold (Au) nanoparticles, can persist in the mononuclear phagocyte system, especially the liver and spleen, posing risks of chronic organotoxicity [154]. Even seemingly biocompatible platforms, such as lipid-based nanoparticles, have encountered regulatory hurdles due to unforeseen proinflammatory responses or “pseudo-allergies” induced by surfactants or lipid oxidation products [155]. These events can exacerbate the microenvironment they aim to modulate, occasionally leading to trial suspension [156].

Variability in nanomaterial composition, manufacturing, and formulation further complicates reproducibility, quality control, and regulatory assessment [157]. These challenges underscore the need for standardized characterization protocols, comprehensive long-term toxicological studies, and well-defined regulatory pathways, implemented through systematic preclinical validation and interdisciplinary collaboration.

Emerging evidence also points to the scalp microbiome as a key factor in follicular health, where microbial dysbiosis may exacerbate perifollicular inflammation [158,159]. Current nanotechnology strategies largely target hormonal and oxidative pathways; however, developing microbiome-responsive nanosystems that selectively modulate the scalp microbial landscape represents a promising frontier for personalized AGA therapy [160].

## 7. Clinical Translation and Future Perspectives

While the reported preclinical efficacy—often exceeding 90% hair follicle recovery in rodent models—is highly encouraging, these metrics must be interpreted with caution. Rodent models, such as C57BL/6 mice [106,107], possess a highly synchronized hair cycle and a thinner dermis, which inherently overestimate the penetration efficiency and therapeutic impact of nanomaterials. Unlike the mosaic growth pattern and deep-seated follicular bulbs (3–5 mm) of the human scalp, rodent follicles are superficial and more accessible to topical nanosystems. Consequently, rapid regeneration in mice may reflect an accelerated telogen-to-anagen transition rather than a true reversal of androgen-driven follicular miniaturization. To bridge this gap, current research is pivoting toward human hair follicle organoids and ex vivo scalp skin models to provide more clinically relevant data (Table 2).

**Table 2:** Translational status and challenges of nanotechnology-based AGA therapies.

Translational Aspect	Current Status and Challenges	Proposed Future Directions	References
Safety and Toxicology	Potential for long-term accumulation of non-biodegradable NPs in the skin; limited systemic toxicity data.	Extensive chronic toxicity studies and use of biodegradable, “green” nanomaterials.	[161]
Manufacturing Scale-up	Batch-to-batch variability; high cost of specialized equipment for complex nanostructures.	Development of microfluidic-based synthesis and standardized manufacturing protocols (GMP).	[162]
Regulatory Hurdles	Lack of specific FDA/EMA guidelines for “nano-cosmeceuticals” and complex delivery systems.	Harmonization of international testing standards; close collaboration with regulatory agencies.	[163]
Clinical Validation	Most data derived from rodent models; human scalp skin thickness and follicle density differ.	Use of 3D-printed human skin models and humanized mice for more accurate preclinical screening.	[164]
Patient Compliance	High-frequency application for topical nanosystems; cost of microneedle-based therapies.	Designing long-acting (e.g., monthly) delivery platforms and low-cost MN manufacturing techniques.	[165]

Despite these challenges, the clinical viability of nanotechnology in dermatology is substantiated by successful applications in related fields, such as nanocrystalline silver for wound healing and lipid nanoparticles for psoriasis. Within the specific context of AGA, the transition from experimental innovation to clinical validation is already underway. As synthesized in Table 3 and Table 4, recent patent disclosures and clinical trials reveal a convergence toward integrated platforms—including exosomes, lipid-based carriers, and microneedle systems. Notably, formulations such as liposomal finasteride (e.g., NCT04574102) and MSC-derived exosomes have successfully achieved therapeutic drug concentrations in human follicles while significantly mitigating systemic exposure. To navigate the associated regulatory landscapes, nano-formulations must be categorized by their intended use: nano-cosmetics target non-living hair fibers for esthetic enhancement; nano-cosmeceuticals are “borderline” products containing bioactive components that influence follicle physiology under less stringent cosmetic regulations; and nano-pharmaceuticals are disease-oriented systems requiring rigorous Phase I–III validation and adherence to FDA/EMA standards. Clarifying these distinctions is essential for aligning nanomaterial design with specific clinical and regulatory objectives.

However, broad clinical adoption still faces hurdles in scalable manufacturing, cost-effectiveness, and regulatory complexity [166]. Future research is expected to focus on stimuli-responsive materials, integration with wearable or light-activated devices, and AI-assisted design. Furthermore, personalized nanomedicine approaches tailored to individual follicular microenvironments and scalp microbiome profiles are poised to enhance therapeutic precision and patient satisfaction, ultimately defining the next generation of AGA management.

### 7.1. Patent Landscape for Nanotechnology-Based AGA Therapy

The increasing commercial interest in nanotechnology-based hair regrowth solutions is reflected in the diversifying patent landscape, as sumarized in Table 3. Current intellectual property disclosures reveal a strategic shift from simple drug encapsulation toward sophisticated, multi-functional delivery platforms. For instance, recent patents highlight the integration of dissolvable microneedles with lipid-based nanocarriers, designed to overcome the physical barrier of the stratum corneum while ensuring the sustained release of growth factors or anti-androgenic agents directly into the follicular niche.

Furthermore, the patent data underscores a rising trend in bio-inspired systems, particularly those involving exosome-mimetic vesicles and bimetallic nanozymes. These disclosures often focus on unique stabilizing formulations or specific nanoparticle-to-ligand ratios that optimize the scavenging of ROS or the modulation of the Wnt/β-catenin pathway [60]. By protecting specific physicochemical properties—such as precise particle size distributions and surface charge modifications—these patents establish the technical foundations for scaling up manufacturing [166]. Ultimately, the transition from broad-spectrum disclosures to targeted, mechanistically driven patents in Table 3 signifies the growing maturity of nanotechnology in the competitive AGA therapeutic market.

### 7.2. Clinical Trials Progress of Nanotechnology-Based AGA Therapies

As evidenced by the clinical trial progress summarized in Table 4, nanotechnology-based hair loss therapies are undergoing a qualitative leap from “laboratory research” to “clinical translation”. These clinical investigations not only validate the high-efficiency delivery capabilities observed in laboratory settings but also confirm the significant advantages of nanoplatforms in enhancing drug bioavailability and reducing systemic side effects within the human environment.

Currently, the focus of clinical translation has shifted from the simple nano-encapsulation of single conventional drugs (such as MXD or finasteride) toward more sophisticated advanced therapies, particularly the synergistic application of exosomes (natural nanovesicles) and microneedle systems. This trend reflects a clinical endorsement of the “microenvironment remodeling” concept: directly regulating oxidative stress, inflammatory status, and angiogenesis around the hair follicle through nano-scale bioactive substances to achieve more sustained hair growth effects than single-agent administration. However, despite the encouraging preliminary results from multiple trials in Table 4, large-scale clinical adoption still faces challenges regarding Good Manufacturing Practice (GMP) standardization, long-term safety monitoring, and the clarification of regulatory classifications. In the future, as more data from Phase III clinical trials are disclosed, nanotechnology is poised to break the deadlock of inconsistent efficacy and poor compliance associated with traditional drugs, driving AGA treatment into a new era of precision medicine and programmed delivery.

Furthermore, as summarized in the clinical trial landscape in Table 4, current investigations of nanotechnology-based therapies for AGA predominantly emphasize the optimization of localized follicular delivery. Accordingly, most trials adopt the established gold-standard topical minoxidil (5%) as the primary active comparator, rather than oral treatment regimens. This trial design reflects the fundamental clinical rationale of nanomedicine. By specifically addressing the systemic toxicity and adverse effects associated with oral therapies—such as sexual dysfunction linked to oral finasteride—nanotechnology-based platforms are primarily evaluated as safer, high-efficacy localized alternatives. Although direct head-to-head comparisons with oral regimens remain limited at current clinical stages, accumulating evidence indicates that nanocarrier systems can significantly enhance the therapeutic index, achieving improved hair density at reduced drug concentrations. Collectively, these findings provide a strong clinical justification for the integration of nanotechnology-based delivery systems into emerging frameworks of precision trichology.

**Figure 5:**
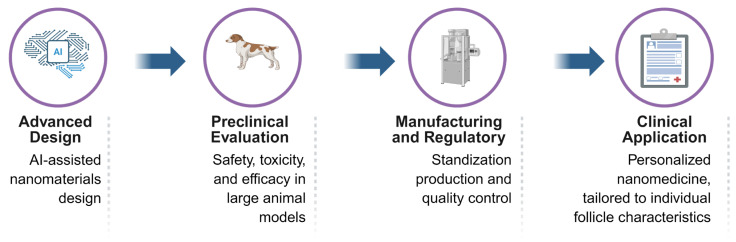
Translational roadmap of nanotechnology-enabled hair regeneration therapies from material design and preclinical evaluation to clinical application and regulatory considerations. Created in Biorender. W. Zhou. (2026) https://app.biorender.com/illustrations/69639a291acd296b13b27700 (accessed on 14 January 2026).

Figure 5 presents a comprehensive translational roadmap for nanotechnology-enabled hair regeneration therapies, outlining the multi-stage progression from laboratory innovation to clinical application. The trajectory begins with the rational design and engineering of nanomaterials, optimizing key physicochemical properties—such as particle size, surface charge, and drug-loading efficiency—to achieve effective follicular targeting and controlled release. This is followed by rigorous preclinical evaluation using both in vitro human hair follicle organoids and in vivo rodent models to assess mechanistic bioactivity, including ROS scavenging, angiogenesis induction, and activation of hair follicle stem cells (HFSCs).

A central component of the roadmap is the critical bridge between safety and scalability. It emphasizes the need for comprehensive toxicological profiling—covering local scalp irritation and systemic bioaccumulation—alongside the development of GMP-compliant manufacturing processes, such as microfluidic-based synthesis for bimetallic nanozymes or exosomes. The pathway culminates in regulatory approval and phased human clinical trials, aimed at establishing long-term efficacy, safety, and patient compliance. By integrating these multidisciplinary milestones, the roadmap provides a strategic blueprint for navigating the “valley of death” in hair loss therapy, ultimately enabling the delivery of standardized, safe, and high-efficacy nanotechnology-based treatments to patients.

## 8. Conclusions

The employment of nanotechnology-based strategies has led to substantial advancements in the treatment of AGA, primarily by facilitating efficient follicular targeting, controlled drug release, and active modulation of the follicular microenvironment. Rather than serving solely as delivery vehicles, emerging nanotechnology-based platforms increasingly address key pathogenic drivers of AGA, including oxidative stress, inflammation, impaired angiogenesis, and stem cell niche dysfunction. However, the future impact of nanotechnology in AGA therapy will depend on the development of more intelligent, mechanism-oriented treatment paradigms rather than incremental improvements in delivery efficiency alone.

In the forthcoming period, it is anticipated that two priority directions will determine the subsequent phase of development in this field. Firstly, the utilization of AI in the design of multi-targeted nanozymes presents a compelling strategy for the engineering of single nanoplatforms capable of simultaneously regulating redox balance, inflammatory signaling and vascular support. This approach aims to more effectively address the multifactorial nature of follicular miniaturization. Secondly, the advent of personalized nano-therapy, predicated on scalp microbiome and niche analysis, portends the imminent realization of precision trichology. In this paradigm, responsive nanosystems are meticulously tailored to individual microbial and inflammatory profiles, thereby ensuring on-demand therapeutic release characterized by enhanced efficacy and safety.

The integration of nanotechnology, AI, and personalized biology offers a compelling framework for the future management of AGA. The translation of next-generation nanotechnology-based therapies into clinical practice will be contingent on sustained interdisciplinary collaboration, complemented by advances in scalable manufacturing and regulatory alignment.

## Figures and Tables

**Table 3:** Representative Patent Disclosures for Nanotechnology-based Hair Regeneration Strategies.

Publication No.	Title/Focus	Key Nanotechnology-Based Strategy	Main Inventive Concept	Status/Region
CN116270562A	Bimetallic nanozyme for oxidative stress-related disorders	Nanozymes (Ni–Cu bimetallic)	Ni–Cu bimetallic nanozyme mimicking endogenous antioxidant enzymes (e.g., SOD/CAT-like activity) to efficiently scavenge ROS and modulate pathological microenvironments relevant to hair follicle degeneration.	CN published
CN113274351B	Liposomal carrier for finasteride with follicular targeting	Liposomes/lipid nanocarriers	Rationally designed liposomal system for finasteride to enhance follicular targeting, local drug retention, and bioavailability while minimizing systemic hormonal exposure.	CN granted
CN117017849A	Exosome-integrated microneedle delivery system	Exosomes + microneedles	Integration of bioactive exosomes into biodegradable microneedle arrays to overcome exosome instability and poor skin penetration, enabling efficient and localized follicular delivery.	CN pending
CN117122552A	Exosome composite microneedle patch for hair regeneration	Exosome-loaded biodegradable microneedle patch	Biodegradable polymer MN embedding exosomes combined with plant-derived bioactives for sustained follicular delivery and hair regeneration.	CN pending
CN112153957B	Use of microneedle patch to promote hair growth	Microneedle arrays with bioactive payloads	Microneedle arrays delivering combinations of natural products (including exosome-related components) and small-molecule growth-promoting agents to stimulate hair growth.	CN active
CN111329832B	Nano-lipid carrier microneedle for hair loss treatment	Lipid nanocarrier-assisted microneedle	Incorporation of nano lipid carriers into microneedles to improve follicular retention and localized delivery of anti-AGA agents (e.g., finasteride).	CN active
US11826461B2	Anti-hair loss core–shell microneedle patch	Core–shell MN with nanozyme + exosomes	Core–shell microneedle architecture co-loading nanozymes and exosomes to simultaneously modulate oxidative stress and activate hair follicle regeneration.	US issued
EP2629782A1/WO2012053976A1	Use of exosomes to promote hair growth	Exosome-based hair growth compositions	Pharmaceutical compositions comprising stem cell-derived exosomes for promoting or enhancing hair growth and wound healing.	EP/WO published
US20210161968A1	Microneedle patch for hair growth (multi-agent)	MN with exosomes + nano-encapsulated agents	Polymeric microneedle arrays incorporating exosomes and nanoparticle-encapsulated small molecules to enhance delivery efficiency and hair growth outcomes.	US application
CN112618572A/CN115252647A	Microneedle-assisted exosome–MXD formulation	Microneedle delivery of exosomes + drug	Microneedle-assisted transdermal delivery of MSC-derived exosomes combined with MXD to enhance lipid metabolism-related hair regeneration.	CN published

**Table 4:** Ongoing or Completed Clinical Trials for Nanotechnology-based and Advanced AGA Therapies.

NCT Number	Phase	Intervention	Nano-Platform/Advanced Strategy	Delivery Method	Study Status
NCT07373054	NA	MXD + Electric Microneedling	Nano-/device-assisted follicular delivery	Automated electric microneedle system	Recruiting
NCT06697080	Phase I/II	hUCMSC-derived exosomes	Natural nano-vesicles for niche modulation	Direct scalp injection	Active, not recruiting
NCT07112586	Phase I/II	Plasma-derived exosomes	Cell-free nano-vesicle therapy	Intradermal scalp injection	Not yet recruiting
NCT06539273	Phase III	Exosome Complex (RNA-loaded)	RNA-carrying exosomal nanoplatform	Topical/local administration	Completed
NCT06239207	Phase II	GFC CELL EXO SCALP KIT	Growth-factor-enriched exosomes	Scalp injection	Completed
NCT06482541	Phase I	Exosomes + microneedling	Exosome-based nano-therapy with physical enhancement	Microneedling-assisted delivery	Not yet recruiting
NCT06551818	NA	SesZen-Bio (Liposomal vs. extract)	Liposomal nano-encapsulation	Topical application	Not yet recruiting
NCT06556056	NA	SesZen-Bio Serum vs. MXD	Liposome-based formulation	Topical application	Completed

## Data Availability

The original contributions presented in this study are included in the article. Further inquiries can be directed to the corresponding authors.

## Abbreviations

The following abbreviations are used in this manuscript:

MXD	Minoxidil
AGA	Androgenetic alopecia
HA	Hyaluronic acid
MN	Microneedle
Shh	Sonic hedgehog
TGF-β	Transforming growth factor-β
BMP	Bone morphogenetic protein
NO	Nitric oxide
ROS	Reactive oxygen species
CAT	Catalase
POD	peroxidase
SOD	Superoxide dismutase
